# The Effect of a Club in Making Differences in Knowledge, Attitude, and Practices on Family Planning Among Married Adolescent Girls in Urban Slums in Bangladesh

**DOI:** 10.3390/ijerph16204037

**Published:** 2019-10-22

**Authors:** Fauzia Akhter Huda, Hassan Rushekh Mahmood, Faisal Ahmmed, Anisuddin Ahmed, Aniqa Tahmina Hassan, Alessio Panza, Ratana Somrongthong

**Affiliations:** 1 College of Public Health Sciences, Chulalongkorn University, Bangkok 10330, Thailand; alessio3108@hotmail.com (A.P.); Ratana.So@chula.ac.th (R.S.); 2 Maternal and Child Health Division (MCHD), icddr,b (International Centre for Diarrhoeal Disease Research, Bangladesh), Dhaka 1212, Bangladesh; hrmahmood@icddrb.org (H.R.M.); faisal.ahmmed@icddrb.org (F.A.); anisuddin@icddrb.org (A.A.); 3 Harvard T. H. Chan School of Public Health, Boston, MA 02115, USA; aniqahassan@hsph.harvard.edu

**Keywords:** married adolescent girls, urban slums, family planning, knowledge, attitude and practices, Bangladesh

## Abstract

Early marriage and childbearing have led to Bangladesh having the highest adolescent fertility rate in the Asia Pacific region. Adolescent pregnancy is correlated with pregnancy-related complications, preterm delivery, delivery of low-birth weight babies, and spousal violence. A quasi-experimental study was conducted in four urban slums (two intervention and two control areas) of Dhaka from July 2014 to August 2016 to assess the effectiveness of a married adolescent girls club (MAG club) in reducing the unmet need for family planning (FP) among married girls between the ages of 14 and 19 (*n* = 1601, 799 in intervention and 802 in control areas). The percentages of the targeted population using any modern method of contraception were significantly higher among respondents in the intervention areas than those in the control areas (72.6% versus 63.5%). The unmet need for FP was significantly lower among respondents in the intervention areas than that of the control areas (16.2% versus 20.7%). The MAG club was a well-received strategy to provide comprehensive information on FP, which in turn helped improve contraceptive method practices and reduced the unmet need for FP among married adolescent girls in urban slums in Bangladesh. The government could leverage its existing resources to expand the MAG Club model in rural parts of the country to achieve the targets outlined in its Adolescent Reproductive Health Strategy.

## 1. Introduction

With a fertility rate of 128 births per 1000 women between the ages of 15 and 19, Bangladesh has the highest adolescent fertility rate in the Asia Pacific [1]. Despite the legal age of marriage being 18 years old for girls, 59% of adolescent girls are married at a younger age [2]. Once married, the girls are often pressured by family members to prove their fertility, which results in early, undesired pregnancies [3].

Adolescent pregnancy is correlated with pregnancy-related complications, preterm delivery, low-birth weight babies, and spousal violence [4]. A higher proportion (19%) of unintended pregnancies (UP) result in abortion when conceived within one year of marriage [5]. The broader social consequences for these girls include lower educational attainment, limited ability to partake in income-generating activities, higher overall fertility rates, and marital and familial difficulties [4].

Evidence shows that married adolescent girls (MAGs) in urban slums are almost twice as likely to be mothers as their counterparts from non-slum areas [5]. Greater than 50% of the pregnancies in slums are unintended, which is more than two-fold greater than the number of unintended pregnancies in MAGs in all urban (22%) and rural (24%) areas of the country [5,6]. These trends align with the finding that the proportion of MAGs in slums with an unmet need for FP was higher (15%) when compared with their counterparts in non-slum urban areas of Bangladesh (10%) [7]. Unintended pregnancies among MAGs in urban slums are largely attributable to the lack of accessible family planning (FP) and reproductive health (RH) services, and non-use or discontinuation of modern contraceptive methods [6,8]. The latter is caused by user-related factors, which includes a lack of awareness of available methods, inconsistent use of methods, fear of side effects, method failure, external pressure from husbands and in-laws to have children, and a lack of spousal communication [6,8]. Findings show that 15% of the MAGs in slums have never used any modern contraceptive method, while 27% have discontinued their chosen method [5].

Although research has been done on contraceptive method use and the unmet need for FP among women of reproductive age in Bangladesh, there are limited data on the specific issues among MAGs in urban slums. More specifically, there are few studies on targeted interventions for this population. Evidence shows that providing comprehensive information on FP through peers and providing innovative behavior change communication (BCC) materials can be effective tools for women in low- and middle-income countries (LMIC) for delivering customized information on and addressing cultural and social barriers to adopting FP services [9].

This study assessed the effectiveness of married adolescent girls’ clubs (MAG clubs) in providing comprehensive information on FP through examining the attitudes, knowledge, and practices of MAGs in urban slums of Dhaka. The expected outcome of this study was to find a difference in the utilization of FP services and methods between the study participants in the control and intervention areas.

## 2. Materials and Methods

### 2.1. Study Setting

The study was implemented in four urban slums of northern and southern parts of Dhaka City Corporation, Bangladesh. These slums were selected because they had definite geopolitical boundaries and were at a distance from each other, preventing the overlap of slum populations and consequent interactions which might bias the evaluation of the intervention. Two of the slums (Mirpur and Kamrangirchar) were randomly assigned to the intervention group and the other two (Shekhertek and Rayerbazar) to the control group. The assignment of the slums between the groups was completed independently by a statistician based on a computer-generated random sequence. It was anticipated that the random assignment of slums would ensure exchangeability between the control and intervention groups.

### 2.2. Study Design

This was a quasi-experimental study with a post-test only design conducted between July 2014 and August 2016, with a 12-month implementation phase lasting from March 2015 to February 2016. Logistical constraints prevented baseline data collection, leading to the post-test only design. To assess the effectiveness of the intervention, a quantitative survey was administered in both the intervention and control areas after the program was completed. Participation in the intervention was open, which meant that study participants were able to join and leave throughout the intervention period.

### 2.3. Study Population

The study population was married adolescent girls (MAGs) between 14 and 19 years’ old who resided in the selected four slums. Both pregnant and non-pregnant MAGs who had lived with their husbands and/or families in the selected slums for at least six months and were willing to participate in the study by providing written consent were included in the intervention. MAGs who were divorced, separated from their spouses, or had speech or hearing disabilities were excluded from the study.

### 2.4. The Intervention

The Government of Bangladesh and local non-governmental organizations (NGOs) run several adolescent clubs in both rural and urban areas of the country. The clubs provide adolescent girls and boys with an opportunity to gather and discuss issues that matter to them, such as birth registration, early marriage, dowry, human rights, sexual and reproductive health (SRH) related issues, including the transmission of STIs, and substance use/drug abuse. The majority of these pre-existing clubs target unmarried adolescent boys and girls, failing to reach adolescent girls who are married. Girls married at an early age may lack access to appropriate SRH information provided by these clubs. To fill this gap, icddr,b, a Bangladesh-based international health research institute that strives to solve key public health problems through high-quality scientific research and innovation, initiated this project through collaboration with brac, an international NGO. brac has been delivering basic maternal, neonatal, and child health (MNCH) and family planning services through a community-based health intervention under its Essential Health Care (EHC) Program, in selected urban slums of several city corporations and selected municipalities, including Dhaka in Bangladesh. Since 2007, this community-based health program, known as Manoshi, has been working to reduce maternal and child mortality in urban slums of Bangladesh by providing normal safe delivery services through its birthing huts at the community level. Family planning (FP) is one of the integrated components of the Manoshi programme and brac has been implementing a few approaches among the eligible couples, women, family members, and community groups to promote FP in addition to maternal, neonatal, and child health care.

Brac has significant experience in running clubs for married and/or unmarried adolescent boys and/or girls in Bangladesh. In this study, brac’s club model was replicated to target married adolescent girls in urban slums. Facilitators to conduct the club sessions were recruited from current brac club leaders as well as brac school teachers. They were trained on matters relevant to SRH, early marriage, unintended pregnancy, and family planning. A total of 10 facilitators were recruited. A team of experts in adolescent and maternal health from icddr,b and brac worked closely to finalize club locations, infrastructure, topics for club sessions, training manuals, and guidelines.

Each month of the intervention had a unique club session curriculum. The 10 facilitators held sessions at their assigned brac school using the pre-established monthly curriculum once every week. There was a total of 40 sessions covering the same material every month. The frequency of sessions allowed for MAGs to have flexibility in selecting a session to attend each month without missing information. Each MAG could attend up to 12 unique club sessions during the one-year intervention period; an average of 20 MAGs participated in each session. A total of 1709 different girls participated in these clubs throughout the intervention period.

Every three months, club participants completed an assessment on the topics discussed during sessions and were awarded token prizes based on their performance for further motivation to remain actively engaged.

Beyond discussion, recreational activities such as dance, music, and drama were arranged for club members. Participants produced and performed plays based on members’ life stories during the final club session. In addition to participating in organized educational and recreational activities, each club attendee received a small pocketbook with information on SRH, early marriage, and family planning in Bengali. This miniature book was designed to be attractive to and practical for teenagers. Each cover was adorned with a mirror and decorative jewels and every book was attached to a key chain.

### 2.5. Sampling Procedure and Sample Size

The survey participants were selected from both the intervention and control areas using a cluster sampling procedure. One kilometer radius from the central point of the slums was denoted as a cluster, and each selected slum was divided into numbers of concentric clusters. A total of 20 clusters were generated for the intervention areas and 15 clusters were generated for the control areas. Then, 10 clusters from each intervention and control areas were selected as study sites through simple random sampling using a computer-generated random sequence. A computer programmer, with no additional involvement in the study, completed the randomization process to prevent bias.

Approximately 6000 households from the 10 clusters of the intervention areas were approached and around 3528 MAGs identified through these household visits were invited to participate in the clubs. Ultimately, 1709 of the MAGs enrolled in the club sessions. Of these participants, 800 were randomly selected for participation in the survey. In the control areas, a random direction was selected from a central location of the slum to determine the first household invited to participate in the quantitative survey. All households in the direction selected were visited and each eligible respondent was invited to take part in the survey. This process was continued until the required sample size was achieved.

In total, 1601 MAGs were included in the survey; 799 from the intervention areas and 802 from the control areas. Each survey was administered through individual face-to-face interviews in both the intervention and control areas. Written consent was gathered from participants before interviews were conducted.

### 2.6. Statistical Analysis

Data were entered into a computer database using Oracle 11G.R2 (Oracle Corporation, Redwood Shores, CA, USA) and exported to STATA 13.1 (Stata, College Station, TX, USA) for analysis. Descriptive statistics, Pearson’s Chi-Square tests, two-sample independent *t*-tests, two-sample proportion test, and Fisher’s exact test were used for quantitative data analysis. Differences in participant characteristics, as well as key outcome indicators between the intervention and control areas, were analyzed for statistical significance at an alpha level of 0.05.

As there were statistically significant differences between study participants from intervention and control areas in socio-economic factors as well as reproductive history, multivariate regression models were developed to control for these differences. However, to investigate the effect of the MAG club in the intervention areas compared with control areas, the logistic regression models were analyzed. Crude and adjusted odds ratios were calculated through separate logistic regression models for each of the following key outcomes: (i) knowledge of modern FP methods; (ii) currently using a modern method of FP; (iii) respondent supports using FP methods; (iv) husband supports using FP methods; (v) responsibility of FP is shared between husbands and wives; (vi) identifies consequences of early pregnancy correctly; and (vii) discussed FP methods with husband. A cluster’s effect was also adjusted during the running of the models. Logistic regression analyses were used to determine if differences in estimating variables in the intervention areas compared with the control areas were statistically significant when adjusted for independent variables. The analyses for these outcomes were all controlled for the educational attainment of both respondents and their husbands, employment status of the respondents, age of marriage, mean duration of marriage in years, and number of pregnancies that the participant has experienced (including current pregnancy). To identify other potential sources of bias, each background characteristic, even those that were not significantly different between intervention and control areas, were logistically regressed with each outcome variable. Additional covariates for each outcome’s regression model were added in a stepwise fashion after controlling for differences in intervention and comparison areas.

### 2.7. Ethical Clearance

The study obtained ethical approval from the institutional review board (IRB) of icddr,b (International Centre for Diarrhoeal Disease Research, Bangladesh) and the Population Council, New York. The Population Council funded this study.

## 3. Results

### 3.1. Socio-Demographic Characteristics of the Respondents and Their Husbands

Among the participants from both the intervention and control areas, 73% were above the age of 18, 25% were 15–17, and the remaining were younger than 15 (Table 1). The majority (98%) of respondents were Muslims. Approximately 14% of the respondents in the intervention areas and 10.8% in the control areas received no formal education. The largest percentage of respondents in both the intervention (40.4%) and control (41.2%) areas had only primary level education. The second largest group received six–eight years of education (31.9% versus 28.9%); a small percentage of respondents received more than nine years of education (13.8 versus 19.2%). One-third (32.3%) of the respondents in the intervention areas were involved in income-generating activities, which was greater than the percentage participating in these activities (14.8%) in the control areas. Of the respondents who generated income, a quarter in both the intervention and control areas worked in garment factories. Half of the respondents in the intervention areas and 10.9% in the control areas were involved in handicrafts. A small proportion of the respondents in the intervention areas (10.5%) and a much higher proportion (41.2%) in the control areas worked as housemaids (Table 1).

The mean age of the husbands of the respondents in both the intervention and control areas was 25. More (23.9%) husbands in the intervention areas had no formal education than those in the control areas (16.7%). One-third of the respondents’ husbands in both areas had received only primary level education. The number of husbands with more than nine years of education was lower in the intervention areas (18.4%) than that in the control areas (25.6%) (Table 1).

A larger percentage of husbands in the intervention areas was involved in garments work (27.3%) than those of the control areas (8.2%). This was followed by a driver or his assistant, business, skilled labor, NGO worker, day laborer, restaurant worker, tailor, and other services in both areas. In both intervention and control areas, the average monthly family income of the respondents was 15,000 Bangladeshi taka (equivalent to 176 USD) (Table 1).

### 3.2. Marriage and Reproductive Characteristics of the Respondents

The majority of respondents in both areas were married before the age of 18 (>90.0%). The mean number of years married was 3.9 (±2.1) in the intervention areas and 3.0 (±2.1) in the control areas. Approximately half of the respondents chose their spouses, while the other half married individuals selected by their parents (Table 2). More than half of the respondents in both areas already had a single pregnancy; 30.8% of the respondents in the intervention areas and 18.6% in the control areas had a history of two or more pregnancies. However, at the time interviews were conducted for this study, fewer respondents in the intervention areas (11.5%) were pregnant than respondents in the control areas (17.3%) (Table 2).

### 3.3. Knowledge and Attitude of the Respondents Towards Contraceptive Method Use, and Consequences of Early Pregnancy

Almost all respondents in both areas were aware of the existence and proper usage of oral pills for contraception. Statistically significant differences were observed in knowledge on other contraceptive methods, including condoms (95.4% versus 66.4%), injection (98.3% versus 89.5%), implant (89.5% versus 56.0%), intrauterine device (IUD) (62.8% versus 12.3%), tubectomy (33.9% versus 8.0%), vasectomy (19.8% versus 4.8%), and the emergency contraceptive pill (17.7% versus 12.3%) in the intervention and control areas, respectively. Knowledge on the consequences of early pregnancy was significantly higher among respondents in the intervention areas (Table 3).

A small number of MAGs in the intervention areas (0.9%) and 4.5% respondents in the control areas did not support using any method of contraception. A small proportion of the respondents’ husbands in the intervention areas (1.4%) and a comparatively higher proportion (4.8%) in the control areas opposed using contraceptive methods. Around three-fourths (72.7%) of the respondents in the intervention areas and less than half (45.9%) of the respondents in the control areas perceived that contraceptive method use was a joint responsibility of both husbands and wives (Table 3).

### 3.4. Family Planning Method Practices and Unmet Need for FP

Higher proportions (72.6%) of respondents in the intervention areas were using modern contraceptive methods (excludes currently pregnant and/or breastfeeding women) when compared with respondents in the control areas (63.5%). A larger number of respondents in the intervention areas than in the control areas used contraceptives, aside from the oral pill which was used at similar rates in both the areas (Table 4).

Respondents who wanted to limit childbearing or delay their next birth by two or more years and were not using any contraceptive methods were considered to have an unmet need for FP. A significant difference in the unmet need for FP was observed among respondents in the intervention areas than that of the control areas (16.2% versus 20.7%) (Table 4).

### 3.5. Participation of Respondents’ in MAG Clubs as a Predictor of Key Outcomes

Table 5 presents the multivariate results of the key outcomes by the selected independent variables. After adjusting for the covariates in the models, the intervention was found to have positively affected the knowledge and practices of FP methods among the MAGs in the intervention areas compared with the MAGs in the control areas.

The adjusted odds of knowing the modern methods of FP and the consequences of early pregnancy was 15.3 times (Adj. OR: 15.3) and 3.4 times (Adj. OR: 3.4) higher in the intervention areas than in the control areas, respectively. Both the adjusted odds ratios were statistically significant, with *p*-values less than 0.005 (Table 5).

The adjusted odds ratio of using a modern FP method was 1.8 (Adj. OR: 1.76), indicating that the likelihood of using a modern FP method was higher in the intervention areas than in the control areas. The odds of participants believing FP is the joint responsibility of both husbands and wives in the intervention areas was 3.4 times (Adj. OR: 3.42) higher than in the control areas when holding all other covariates constant (Table 5)

## 4. Discussion

This quasi-experimental study was designed to assess the effectiveness of an innovative intervention, the married adolescent girls’ club (MAG club), to see whether there were differences in the knowledge of, attitude towards, and utilization of contraceptive method practices among two groups (intervention and control) of married adolescent girls in urban slums of Dhaka, Bangladesh.

It was revealed that a noteworthy number of married adolescent girls had received extensive information on family planning from the MAG clubs. More respondents in the control areas than the intervention areas were pregnant at the time of evaluation, suggesting that the MAG club may have had a positive influence in reducing the number of pregnancies.

Findings from this study showed that other than the oral pill, there were significant differences in the knowledge of different contraceptive methods and of potential problems associated with adolescent pregnancy between the groups. Respondents in the intervention areas with no children expressed their intention to delay their first pregnancy by at least three to five years, an observation not found in the control areas. The current study also revealed a significant difference in attitudes towards contraceptive method use among respondents of the two groups; more respondents and their husbands in the control areas did not support any FP method use than their counterparts in the intervention areas. Furthermore, a larger percentage of respondents and husbands in the intervention areas indicated that family planning was a shared responsibility. After controlling for respondents’ characteristics that were different between control and intervention areas, the adjusted odds of believing FP is a shared responsibility were still higher in the intervention areas than the control areas. This suggests an association between the intervention and the outcome that was still present after controlling for potential sources of bias.

Findings from another study revealed husbands’ opposition to contraception as a barrier to the use of FP methods, even if women had the knowledge, access, and intention to use contraceptives [10]. However, the current study identified that respondents in the intervention areas discussed FP methods with their husbands, which could be the effect of an element of the sessions that taught women how to engage their husbands in family planning discussions and decision making. Findings also showed comparatively greater use of male methods (condoms) in the intervention areas, which is suggestive of one of several key elements of empowerment and gender norm change found in other studies [11].

The current use of any modern contraceptive methods and its adjusted odds were higher and the unmet need of family planning was lower among respondents in the intervention areas than in the control areas. This finding is in alignment with research utilizing data from BDHS 2004, which found that having an unmet need was significantly lower (*p* < 0.001) when there was spousal communication about contraceptive methods [12]. Long-acting reversible contraceptive (LARC) methods uptake in the intervention areas was higher than that of the control areas, suggesting that there may be an association between club participation and receptiveness towards this method. A similar finding was observed in a study in Malawi on peer leaders trained to deliver youth-friendly FP services [13].

### 4.1. Strengths of the Study

Although several programs to improve the SRH of adolescents are being implemented in Bangladesh, the efficacy of these interventions is rarely documented. Furthermore, there is a lack of programs focused exclusively on married adolescent girls in urban slums. This is the first study to document the effectiveness of MAG clubs in generating knowledge on, changing the attitude towards, and encouraging practices of family planning among married adolescent girls in urban slums. Additionally, the presence of a control group in this quasi-experimental design acted as a proxy for the counterfactual and partially mitigates history and maturation threats. As the same survey was used for all participants, testing and instrumentation threats were controlled.

### 4.2. Limitations of the Study

Although a quasi-experimental study, the post-test only design limits the conclusions that can be drawn from the data. As there was no pre-test data, the differences observed between intervention and control groups cannot be attributed solely to the program. Furthermore, there were differences between the intervention groups and the control groups, such as employment, that may have affected internal validity; however, the logistic regressions revealed significant results even after controlling for these factors. There were still factors that could not be controlled. For example, as participants in the intervention areas chose to join MAG clubs and assignment to the intervention was not randomized, selection bias may have threatened internal validity. Recall bias may have resulted from the use of self-reported data. There may be confounding factors which were not accounted for and causal inferences cannot be made. As participants were not involved in the design of the FP pocketbook, the acceptability and utility of this element of the intervention among participants is not certain. Furthermore, findings of this study may not be generalized to married adolescent girls living in rural areas of Bangladesh, as the adolescents living in those areas have different profiles and living conditions.

## 5. Conclusions

Adolescent pregnancy is a major social and health concern in Bangladesh. An appropriate intervention for married adolescent girls must be implemented to increase access to correct and comprehensive information on FP methods and services. Early marriage is a central component to the physical, social, and economic risks of early pregnancy, and girls from urban slums are especially vulnerable. Along with FP, the deleterious effects of early marriage, early pregnancy, and motherhood are important messages that need to be conveyed to this group of young women [14]. The findings of this intervention suggest that the married adolescent girls’ club (MAG Club) was an effective and well-received strategy to generate knowledge on and encourage the use of contraceptive methods. MAG clubs have the potential to effectively improve married adolescents’ sexual and reproductive health (SRH) in urban slums in Bangladesh.

The current Strategic Plan for Health, Population, and Nutrition Sector Development Programme (HPNSDP) [15] encourages the adoption of long-acting and permanent methods (LAPM) of contraception, as these methods are used minimally throughout the country. In addition, the Government of Bangladesh has placed an emphasis on improving the health of adolescents through developing the National Adolescent Health Strategy [14]. To meet the targets in this strategy, the government could leverage its existing resources and workforce in urban areas, specifically in the slums, to replicate the club model and engage with this age group more efficiently. Capacity building of the community health workers, through collaboration between the government and the NGOs, could further improve family planning service provision and uptake. This would allow for the government to not only focus on adolescents but encourage their use of methods in alignment with strategic policy.

Further research is required to ascertain whether statistically significant differences between the intervention and control areas are attributable to MAG clubs. Additionally, this intervention should be studied in rural settings of Bangladesh to assess its feasibility and which adaptations are necessary for replication and scale-up nationally. This can be accomplished by utilizing the government’s existing infrastructure and resources allocated to prevent adolescent motherhood.

## Figures and Tables

**Table 1:** Sociodemographic characteristics of the respondents and their husbands by intervention and control areas.

Characteristics	Respondents *n* (%)		*p*-Value
	Intervention (*n* = 799)	Control (*n* = 802)	
Respondents’ age group (in Years)			
<15	3 (0.4)	11 (1.4)	0.092
15–17	211 (26.4)	202 (25.2)	
≥18	585 (73.2)	589 (73.4)	
Respondents’ religion **			
Muslim	781 (97.7)	789 (98.4)	0.359
Others	18 (2.3)	13 (1.6)	
Respondents’ education (in completed years) **			
No education	111 (13.9)	86 (10.8)	0.009*
1–5	323 (40.4)	329 (41.2)	
6–8	255 (31.9)	232 (28.9)	
9+	110 (13.8)	155 (19.2)	
Respondents’ occupation **			
Involved in income generation	258 (32.3)	119 (14.8)	0.000 *
Not involved	541 (67.7)	683 (85.2)	
Types of respondents’ occupation **	N = 258	N = 119	
Housemaid	27 (10.5)	49 (41.2)	0.000 *
Garment/factory worker	53 (20.5)	30 (25.2)	
Handicrafts	134 (51.9)	13 (10.9)	
Other services	44 (17.1)	27 (22.7)	
Respondents’ husbands’ age (in years)			
Mean (±sd) ^†^	25.3 (3.51)	24.9 (4.05)	0.072
Respondents’ husbands’ education (in completed years) **			
No education	191 (23.9)	134 (16.7)	0.000 *
1–5	273 (34.2)	249 (31.0)	
6–8	188 (23.5)	214 (26.7)	
9+	147 (18.4)	205 (25.6)	
Types of respondents’ husbands’ occupation **			
Garments/factory worker	218 (27.3)	66 (8.2)	
Bus/car driver/assistant to driver	182 (22.8)	247 (30.8)	
Business	110 (13.8)	131 (16.3)	
Skilled labor	65 (8.1)	105 (13.1)	
Non-government service	63 (7.9)	118 (14.7)	0.000 *
Day laborer	61 (7.6)	58 (7.2)	
Works in restaurant/shop	38 (4.8)	20 (2.5)	
Tailor	23 (2.9)	16 (2.0)	
Others	39 (4.9)	41 (5.2)	
Monthly family income (in BDT) ***			
Mean (±sd) ^†^	15,513 (±9423)	15,208 (±8727)	0.501

* Significant difference; § *p*-value was calculated using Fisher’s exact test; ** *p*-value was calculated using chi-square test; ^†^ *p*-value was calculated using two sample mean test (independent *t*-test); *** 1 USD was equivalent to 83 Bangladeshi taka (BDT).

**Table 2:** Marriage and reproductive characteristics of the respondents by intervention and control areas.

Characteristics	Respondents’ *n* (%)		*p*-Value
	Intervention (*n* = 799)	Control (*n* = 802)	
Respondents’ age at marriage (in years) **			
≤14	383 (47.9)	277 (34.5)	0.000 *
15–17	391 (48.9)	445 (55.5)	
≥18	25 (3.1)	80 (10.0)	
Mean (±sd) †	14.6 (±1.6)	15.2 (±1.8)	0.000 *
Duration of marriage (in years)			
Mean (±sd) †	3.9 (±2.1)	3.0 (±2.1)	0.000 *
Type of marriage **			
Marriage by own choice	387 (48.4)	329 (41.0)	
Marriage by parents’ choice	412 (51.6)	473 (59.0)	0.003 *
Pregnancy history **			
No pregnancy history	104 (13.0)	188 (23.4)	
Single pregnancy	449 (56.2)	465 (58.0)	0.000 *
≥ 2 pregnancy	246 (30.8)	149 (18.6)	
Current pregnancy status **			
Pregnant	92 (11.5)	139 (17.3)	
Not pregnant	707 (88.5)	663 (82.7)	0.001 *

* Significant difference; ** *p*-value was calculated using the chi-square test; † *p*-value was calculated using two-sample mean test (independent *t*-test).

**Table 3:** Knowledge and attitude differences of the respondents of family planning methods and the consequences of early pregnancy by intervention and control areas.

Characteristics	Respondents’ *n* (%)		*p*-Value
	Intervention (*n* = 799)	Control (*n* = 802)	
Knowledge about FP methods ^#^	*n* = 799	*n* = 798	
Oral pill ‡	794 (99.4)	795 (99.6)	0.479
Injection ‡	785 (98.3)	714 (89.5)	0.000 *
Condom ‡	762 (95.4)	530 (66.4)	0.000 *
Implant ‡	715 (89.5)	447 (56.0)	0.000 *
IUD ‡	502 (62.8)	98 (12.3)	0.000 *
Tubectomy ‡	271 (33.9)	64 (8.0)	0.000 *
Vasectomy ‡	158 (19.8)	38 (4.8)	0.000 *
Emergency contraceptive pill ‡	141 (17.7)	98 (12.3)	0.003
Knowledge of the consequences of early pregnancy ^#^			
Health risk of mother ‡	753 (94.2)	680 (84.8)	0.000 *
Health risk of baby ‡	619 (77.5)	242 (30.2)	0.000 *
Maternal death ‡	405 (50.7)	315 (39.3)	0.000 *
Neonatal death ‡	282 (35.3)	222 (27.7)	0.001 *
More chances of caesarean delivery ‡	105 (13.1)	161 (20.1)	0.000 *
More chances of having miscarriages ‡	103 (12.9)	16 (2.0)	0.000 *
Attitude on support for using FP methods §			
Supported	792 (99.1)	756 (94.7)	0.000 *
Didn’t support	7 (0.9)	42 (5.3)	
Perceptions of husbands’ attitude for using FP methods §			
Supported	786 (98.4)	728 (91.2)	0.000 *
Didn’t support	11 (1.4)	38 (4.8)	
Didn’t know	2 (0.2)	32 (4.0)	
Perceptions on responsibility of FP method use **	*n* = 799	*n* = 802	
Respondents’ responsibility	139 (17.4)	273 (34.0)	
Husband’s responsibility	79 (9.9)	161 (20.1)	0.000 *
Responsibility of Both	581 (72.7)	368 (45.9)	

^#^ Only the correct answers were calculated, multiple responses; * significant difference; ‡ *p*-value was calculated using two-sample proportion test; ** *p*-value was calculated using the chi-square test; § *p*-value was calculated using Fisher’s exact test.

**Table 4:** Family planning method practices and unmet need for family planning among the respondents by intervention and control areas.

Characteristics	Respondents’ *n* (%)		*p*-Value
	Intervention	Control	
	*n* = 799	*n* = 802	
Ever used family planning methods **			
Used	749 (93.7)	709 (88.4)	0.000 *
Never used	50 (6.3)	93 (11.6)	
Current use of modern FP methods **	*n* = 749	*n* = 709	
Using	544 (72.6)	450 (63.5)	0.000 *
Not using	205 (27.4)	259 (36.5)	
Types of current method use §	*n* = 544	*n* = 450	
Pill	201 (37.0)	210 (46.7)	0.032 *
Injection	195 (35.9)	142 (31.6)	
Condom	100 (18.4)	75 (16.7)	
Implant	31 (5.7)	15 (3.3)	
IUD	12 (2.2)	6 (1.3)	
Sterilization (Male/Female)	5 (0.9)	2 (0.4)	
Unmet need for family planning **	*n* = 799	*n* = 802	
Yes	129 (16.2)	166 (20.7)	0.019 *
No	670 (83.8)	636 (79.3)	

* Significant difference; ** *p*-value was calculated using the chi-square test; § *p*-value was calculated using Fisher’s exact test.

**Table 5:** Effect of the intervention on knowledge and practices of FP methods among the MAGs compared between intervention and control areas.

Characteristics	Adjusted Odds Ratio	*p*-Value for Adjusted OR
Knows of modern methods of contraception		
	15.26175	<0.0001
Consequences of early pregnancy properly identified		
	3.409266	<0.0001
Discussed FP with husband		
	7.447658	<0.0001
Supports using FP		
	6.552657	<0.0001
Husband supports using FP		
	3.955414	<0.0001
Currently using a modern contraceptive method		
	1.769515	0.002
FP is a joint responsibility of husband and wife		
	3.426354	<0.0001
